# Normative Expectations in Human and Nonhuman Animals

**DOI:** 10.1177/17456916231187401

**Published:** 2023-07-28

**Authors:** Susana Monsó, Richard Moore

**Affiliations:** 1 Department of Logic, History, and Philosophy of Science, Universidad Nacional de Educación a Distancia; 2 Messerli Research Institute, Veterinärmedizinische Universität Wien; 3 Department of Philosophy, University of Warwick

Heyes proposes that human normative psychology is not a result of natural selection in the hominin lineage but a learned product of cultural feedback. On this view, norms result from the internalization and subsequent articulation of behavioral-reinforcement strategies.

Heyes’s account contains three elements. First (“compliance”), people act in ways likely to gain others’ approval while avoiding their disapproval. Second (“enforcement”), approved behaviors give rise to positive reactions in people, whereas behaviors that are negatively received engender negative reactions. Third (“commentary”), when considering one’s own and others’ behavior, people make judgments about what is appropriate using normative language. People’s normative psychology is thus the result of cultural learning and the internalization of others’ attempts to constrain their behavior. Explicitly normative processes of deontic reasoning are learned through verbal commentary and build on the implicit processes of enforcement and compliance in which people reward liked behaviors while protesting disliked ones. Compliance and enforcement appear to be individually necessary and together sufficient for the implicitly normative cake, with commentary, which makes norms explicit, the language-involving icing on top. However, Heyes’s view is not entirely clear.

Heyes contrasts her account with work by evolutionary psychologists, who explain norm psychology as a product of recent evolutionary history. According to Tomasello (2022), for example, selective pressures from living in complex social groups made humans’ hominin ancestors undergo a suite of adaptations for “shared intentionality,” including skills and motivations for norm cognition. Tomasello’s hypothesized adaptations purport to explain why only humans are normative animals. We admire Heyes’s attempt to specify the cognitive mechanisms through which normative cognition is expressed and learned without recourse to adaptations. Nonetheless, her view leaves several questions unanswered, including the question of whether there can be normative animals.

On Tomasello’s (2022) view, the absence of normative behaviors in nonhuman species is a consequence of their lacking an evolved norm psychology. Heyes’s more minimal account of the psychological foundations of norm cognition suggests that there may be normative animals because implicit normativity is dependent on a suite of domain-general learning processes that are highly conserved. Whereas nonhumans lack the language needed to offer normative commentary, if enforcement and compliance alone suffice for normativity, there could be nonverbal normative creatures. Alternatively, if commentary is necessary for normativity “proper,” this seems to beg the question against the existence of nonverbal normative agents. This generates a dilemma. Either implicit normativity is already normative, in which case, a wide range of cognitively unsophisticated species would be normative agents, or explicit normativity is what provides normativity. The former seems improbable; the latter is question-begging.

Perhaps Heyes will embrace the first horn of this dilemma. But we worry that this is too quick. Consider chimpanzees. Although there is some evidence that they engage in compliance and enforcement, it is generally weak (for discussion, see Schlingloff & Moore, 2017). There are reports of cultural behaviors that may serve an affiliative function—potentially evidence for compliance. Van Leeuwen and colleagues (2014) described how chimpanzees at Chimfunshi copied a high-ranking female’s practice of putting grass in her ear, and Hobaiter and Byrne (2010) described chimpanzees at Budongo as affectionately copying the unusual gait of a physically disabled peer. There is scant evidence of enforcement, however. Rudolf von Rohr and colleagues (2015) reported that when chimpanzees witness aggression against even unrelated infants, they react by screaming—seemingly a case of protesting unwanted treatment. Nonetheless, chimpanzees seem largely unphased by the ill treatment of unrelated others. When Rudolf von Rohr and colleagues showed chimpanzees videos of conspecific infanticide, they looked longer at videos of unfamiliar individuals committing infanticide than at control videos of chimpanzees behaving aggressively toward adults. However—with the exception of one individual who displayed at the screen—watching infanticide did not elicit negative emotional arousal. It therefore seems possible that although chimpanzees do not appreciate the problematic behaviors of others, they do not care that much. Do these minimal enforcement behaviors deserve the label “normative”?

Given that Heyes calls compliance, enforcement, and commentary “normative behaviors,” perhaps each of them is sufficient for some degree of normativity and chimpanzees qualify as normative agents. But if Heyes is willing to grant normativity wherever there is some degree of conformity based on social learning, does her view also imply that fruit flies are normative creatures? Danchin et al. (2018) showed that when female Drosophila observe the mating preferences of female conspecifics, they subsequently mate with males who look like the one in the demonstration. In effect, these flies “learned to prefer males of a given color” (p. 1026). These preferences spread through the population, giving rise to “local traditions” (p. 1029). Is this enough for normativity? Or does Heyes require the additional layer of enforcement? If so, are compliance and enforcement necessary and cosufficient for normativity, as we originally supposed? Or is the third layer—commentary—necessary for “full-blown” normativity?

Heyes’s discussion of commentary prompts further questions. She suggests that commentary not only relies on the explicit expression of approval and disapproval but also incorporates normative language. This is striking because there could be forms of commentary that refrain from making general claims about permissibility—for example, (a) “I do not like your behavior” rather than the normatively loaded (b) “Your behavior is inappropriate.” In contrast to Example a, Example b introduces an appeal to some kind of objectivity or a plural subject (Tomasello, 2014), the origins of which have yet to be explained. In appealing to normative language, is Heyes thus smuggling in some additional ingredient over and above articulating dissatisfaction? If so, what work is normative language doing for her account? And where does people’s tendency to use this language come from? Here Tomasello can appeal to humans’ evolved norm psychology to explain the emergence of normative talk. Heyes cannot. We think she needs a story about the cultural evolution of normative language that does not assume prior understanding of normative concepts. Moreover, if researchers are to take seriously the question of whether animals are normative creatures, they need a definition of normativity that does not just assume that full-blown normativity is language-dependent.

These issues leave us unsure of whom Heyes counts as normative agents and on what grounds.

## Transparency

*Action Editor:* Daniel Kelly

*Editor:* Interim Editorial Panel
